# Criminal justice measures for economic data harmonization in substance use disorder research

**DOI:** 10.1186/s40352-018-0073-6

**Published:** 2018-09-21

**Authors:** Kathryn E. McCollister, Xuan Yang, Sean M. Murphy, Jared A. Leff, Richard A. Kronmal, Heidi M. Crane, Redonna K. Chandler, Faye S. Taxman, Daniel J. Feaster, Lisa R. Metsch, William E. Cunningham, Frederick L. Altice, Bruce R. Schackman

**Affiliations:** 1 Department Public Health Sciences, University of Miami Miller School of Medicine, Miami, FL USA; 2 Department of Healthcare Policy and Research, Weill Cornell Medicine, New York, NY USA; 3 Collaborative Health Studies Coordinating Center, University of Washington, Seattle, WA USA; 4 Center for AIDS Research, University of Washington, Seattle, WA USA; 5 AIDS Research Program, National Institute on Drug Abuse, Bethesda, MD USA; 6 Center for Advancing Correctional Excellence, George Mason University, Fairfax, VA USA; 7 Department of Sociomedical Sciences, Mailman School of Public Health, Columbia University, New York, NY USA; 8 Department of Medicine, UCLA, Los Angeles, CA USA; 9 Yale AIDS Program, Section of Infectious Diseases, Yale School of Medicine, New Haven, CT USA

**Keywords:** Economic evaluation, Social costs of crime, Data harmonization, Economic outcomes

## Abstract

**Background:**

The consequences of substance use disorders (SUDs) are varied and broad, affecting many sectors of society and the economy. Economic evaluation translates these consequences into dollars to examine the net economic impact of interventions for SUD, and associated conditions such as HCV and HIV. The nexus between substance use and crime makes criminal justice outcomes particularly significant for estimating the economic impact of SUD interventions, and important for data harmonization.

**Methods:**

We compared baseline data collected in six NIDA-funded Seek, Test, Treat and Retain (STTR) intervention studies that enrolled HIV-infected/at-risk individuals with SUDs (total *n* = 3415). Criminal justice measures included contacts with the criminal justice system (e.g., arrests) and criminal offenses. The objective was to develop a list of recommended measures and methods supporting economic data harmonization opportunities in HIV and SUD research, with an initial focus on crime-related outcomes.

**Results:**

Criminal justice contacts and criminal offenses were highly variable across studies. When measures grouped by offense classifications were compared, consistencies across studies emerged. Most individuals report being arrested for property or public order crimes (> 50%); the most commonly reported offenses were prostitution/pimping, larceny/shoplifting, robbery, and household burglary.

**Conclusions:**

We identified four measures that are feasible and appropriate for estimating the economic consequences of SUDs/HIV/HCV: number of arrests, number of convictions, days of incarceration, and times committing criminal offenses, by type of offense. To account for extreme variation, grouping crimes by offense classification or calculating monthly averages per event allows for more meaningful comparisons across studies.

## Background

The consequences of substance use disorders (SUD) are varied and broad, affecting many sectors of society and the economy – personal health, public health (e.g., the spread of infectious diseases), education, workplace, social services, and criminal justice (Balsa et al. 2009; Caulkins and Nicosia 2010; Gamarel et al. 2017; Godfrey et al. 2004; McCollister and French 2003; National Institute on Drug Abuse 2017). Economic evaluation methods quantify the value of resources associated with both an intervention and the disorder, including the monetary value of these multi-sectoral consequences.

A full economic evaluation comprises the comparison of two or more programs (e.g., intervention vs. treatment-as-usual) and must include assessment of both the costs and consequences of each program. Cost-effectiveness analysis (CEA) is one economic evaluation approach, which compares the cost of treatment to its impact on a clinical effectiveness outcome such as substance use, HIV-risk, or mortality. Results are expressed as an incremental cost-effectiveness ratio, which describes the cost to achieve a unit of outcome (e.g., a day of abstinence) in the intervention condition relative to its comparator. CEA does not require the effectiveness measure to be translated into dollars, and it allows for comparisons across a broad range of healthcare interventions as long as they have a common outcome of interest (Neumann et al. 2016; Drummond et al., 2015). CEAs can be framed from the healthcare sector perspective, the perspective of a specific payer, or the broader societal perspective, which incorporates costs outside the healthcare sector such as costs to patients and their families, criminal justice involvement and social services utilization.

A cost-benefit analysis (CBA) compares the cost of treatment to the economic benefits generated from reduced costs to the health sector, other sectors (e.g., criminal justice, education, social services), and to patients/families, as well as increased productivity and earnings (Drummond et al., 2015). To calculate economic benefits, outcomes from these domains must be translated into dollars using monetary conversion factors (e.g., cost per overnight hospital stay, cost per day in transitional housing, cost per day incarcerated). CBA expresses results as net economic benefits (benefit minus cost) and considers an intervention cost-beneficial if the net benefit estimate is positive. CEA and CBA provide economic evidence that complements clinical evidence of intervention effectiveness, as it informs stakeholders about the resources needed to implement an intervention, the return on investment in terms of cost to achieve desired outcomes, and factors such as intervention scalability and sustainability.

The National Institute on Drug Abuse (NIDA) considers data harmonization in research pertaining to substance use, human immunodeficiency virus (HIV), hepatitis C virus (HCV), and other related diseases/disorders a high priority research area (National Institute on Drug Abuse, 2014). Data harmonization initiatives funded through NIDA highlight the importance of having high quality data that can be synthesized to promote more rigorous and generalizable analyses of the impact of an intervention, program, or policy (Chandler et al. 2015; Fortier et al. 2011; Hamilton et al., 2011). The nexus between substance use and crime makes criminal justice outcomes particularly significant for estimating the economic impact of SUD interventions, and important for data harmonization (Anglin and Perrochet 1998; Harrison et al. 2001). Intersections between substance use, HIV, and criminal justice involvement also highlight significant health disparities in the United States. African Americans, in particular, are more likely to be incarcerated and have a higher risk of HIV infection than Whites or Hispanics, sharing disproportionately in the economic and social burden of SUD and HIV (Aral et al. 2008; Binswanger et al. 2012).

NIDA funded a large-scale, prospective data collection and harmonization effort across 22 unique studies testing HIV continuing care interventions for individuals with substance use disorders, referred to collectively as the Seek, Test, Treat, and Retain (STTR) Initiative (Chandler et al., 2017; Chandler et al. 2015). The studies enrolled individuals with a substance use disorder who were either HIV positive or at-risk for HIV and were divided into two groupings – studies focusing on criminal justice populations and studies focusing on vulnerable populations. Within each grouping, STTR investigators worked collaboratively to define core research questions, outcome domains and measures, and data collection and management processes (Chandler et al. 2017; Chandler et al. 2015; Montague et al., 2012; Montague et al., 2011). All studies were expected to adopt the agreed-upon core outcome domains, but they had flexibility to define other outcomes of interest, including measures that can be used for economic analyses. Crime-related measures were not part of a core domain for the STTR studies focusing on vulnerable populations, but many of these studies opted to include questions on arrests, charges, and other contacts with the criminal justice system. The STTR initiative provides an opportunity to examine the potential for harmonization of clinical and economic data that is feasible and appropriate for conducting economic evaluations of interventions for substance use disorders, HIV, and HCV.

In this study, we examined baseline crime-related measures from six STTR studies, to identify outcomes that are similar across studies and conducive to an economic evaluation. Costs associated with criminal activity include tangible costs to the criminal justice system (e.g., police protection, legal and adjudication, incarceration), to victims (e.g., property damage), and to society (e.g., lost productivity), as well as intangible costs to victims (i.e., pain and suffering) (McCollister et al. 2010). In evaluating the net impact of an intervention, failure to account for reductions in criminal activity costs may result in undervaluing it from public payer and societal perspectives. The primary objective of this study was to review commonly collected outcome measures and propose standard measures that can be used for estimating the costs of criminal activity and criminal justice outcomes from different stakeholder perspectives. We consider strategies to harmonize economic data across different studies and assessment timeframes. The implications of translating criminal activity measures into dollars are discussed. Results will inform future study designs for economic evaluations and broaden the scope of economic impact analyses.

## Methods

To gain access to STTR data, we submitted a concept proposal to the STTR Data Coordination Center at the University of Washington, which was reviewed and approved (April 2016). Given the sensitive nature, these data are not publicly available. Individuals interested in collaborating or working with these data should contact the STTR Data Coordination Center at sttr@uw.edu. For our study, we selected six STTR studies based on availability of at least two pre-specified crime-related measures and completeness of baseline data at the time we made our data request.

Table 1 provides an overview of the studies including study objective, population, primary outcome, sample, setting, and timeline. Two of the studies worked with criminal justice populations (BRIGHT 1 and 2); the remaining four studies worked with vulnerable populations (RETAIN, STRIDE 1 and 2, PACTo). Collectively, these studies represent 1010 HIV-infected and 2405 at-risk for HIV individuals. Outcome data are based on self-report by study participants.

**Table 1:** Economic data harmonization in STTR studies - study descriptions

Study	Objective	Population	Primary Outcome(s)	Sample Size	Location	Timeframe^a^
Project RETAIN: Providing Integrated Care for HIV-Infected Crack Cocaine Users	to evaluate the efficacy of an integrated “retention clinic” in achieving virologic suppression compared to treatment as usual	HIV-infected cocaine users	virologic suppression at 12-months	360	HIV clinics in Miami, FL and Atlanta, GA	2013–2017
BRIGHT 1: Baltimore-Rhode Island Get HIV Tested	to determine acceptability of on-site rapid HIV testing versus off-site referral	probationers and parolees not known to be HIV positive	undergoing HIV testing and receipt of HIV testing results	2405	community corrections offices in Baltimore City, MD and Providence / Pawtucket, RI	2011–2016
BRIGHT 2: Baltimore-Rhode Island Get HIV Tested	to determine effectiveness of HIV linkage to care comparing intensive case management to treatment as usual	probationers and parolees with known HIV-infection	HIV treatment engagement and retention	100	community corrections offices in Baltimore City, MD	2011–2015
STRIDE 1: HIV, Buprenorphine, and Criminal Justice	to compare buprenorphine treatment to placebo for opioid dependence treatment	HIV-infected, community-supervised defendants or offenders with opioid dependence	HIV-related outcomes^b^	50	Washington, DC	2012–2018
STRIDE 2: HIV, Buprenorphine, and Criminal Justice	to examine if there are differences in HIV, drug use, and other outcomes among individuals receiving treatment versus individuals actively using, not actively using and not in treatment, and individuals on methadone, Suboxone, or in some other treatment	HIV-infected adults with opioid-dependence eligible for Medicaid or other insurance	HIV-related outcomes^b^	100	Washington, DC	06/2014–10/2014
PACTo: Enhanced Access to HIV Care for Drug Users in San Juan, Puerto Rico	to implement and evaluate a community-level, structured approach, the “Enhanced HIV Care Access and Retention Intervention”	drug users living with HIV	virologic suppression every 6-months for up to 36 months	400	five communities in San Juan, PR	2014–2017

^a^Timeframe is defined as years in which partipiants were first and last enrolled

^b^HIV-related outcomes include viral load, CD4 count, retention in care, HIV risk behaviors

Crime-related measures included contacts with the criminal justice system (arrests, charges, convictions, incarceration) and criminal offenses. We first reviewed measures within each study to identify outcomes that are comparable across studies and could potentially be used for economic analyses. A prerequisite is that variables must represent counts (e.g., number of arrests). Dichotomous measures such as “ever been arrested” or “ever committed an illegal act” cannot be monetized for use in economic evaluations. An additional consideration is that while the baseline costs of criminal activity can serve as an important comparator or predictor of post-intervention costs, to estimate reduced crime costs during an intervention period (attributable to the intervention), baseline and follow-up data are required and should represent equal recall timeframes. For example, comparing the costs of “days incarcerated over lifetime” at baseline and “days incarcerated during past 90 days” at follow-up is not meaningful.

A database of crime/criminal justice measures represented in two or more of the selected STTR studies was constructed, allowing us to proceed with data cleaning, organization, and quality checks. Variables were evaluated in terms of missingness, logical inconsistencies (e.g., reporting an arrest but not reporting the charge), as well as basic coding issues such as invalid character values. Measures were grouped into four categories used by the Bureau of Justice Statistics and FBI Uniform Crime Reporting System: (1) property crimes; (2) violent crimes; (3) public order crimes; and (4) enterprise crimes (Truman and Morgan 2016; U.S. Department of Justice, 2004). Table 2 summarizes the individual offenses included within each crime category organized by offenses with/without monetary conversion factors (MCFs).

**Table 2:** Summary of individual crimes by crime category

Crime Category	Offenses With Monetary Conversion Factors	Offenses Without Monetary Conversion Factors
Property Crimes	Fencing (buying or selling stolen property), burglary/breaking and entering (home, auto, business), larceny, vandalism, property damage, auto theft, carjacking, arson	Shoplifting, tagging
Violent Crimes	Robbery/attempted robbery, mugging, assault, aggravated assault, battery, homicide, manslaughter, attempted homicide, weapons offenses, sexual offenses (rape, sex with minor)	
Public Order Crimes	Prostitution or pimping	Illegal gambling, terrorist threats/acts, probation/parole violations, trespassing, disorderly conduct, contempt of court, drug crimes
Enterprise crimes	Forgery/fraud	Kidnapping/hostage taking

Note: Given the lack of precise costing data for every reported offense, the same MCF could be applied to similar offenses such as auto theft and carjacking, or assault and battery

Baseline criminal activity measures were reported across varying time frames: past 90 days (2 studies), past 6 months (2 studies), and past 30 days (2 studies). To normalize different baseline assessment timeframes for self-reported crimes and incarceration, we created measures of average number of offenses per 30 days and average days incarcerated per 30 days. We considered an alternative approach to adjust data by extrapolating to the longest reference period, which was six months. For instance, “past 90 day” number of times arrested can be multiplied by two to represent a “past 6 month” timeframe. Both adjustments rely on a limiting assumption that the rate of arrest or offending remains constant over time; although, creating an average with real data points vs. adding data points through extrapolation was deemed a more conservative and therefore preferred approach.

Descriptive statistics were constructed for each study. Given the importance placed on arithmetic mean values in economic evaluations in order to draw policy-relevant conclusions for population health (Neumann et al. 2016), a one-way analysis of variance (ANOVA) was performed to assess differences across studies. Due to the long right tail on the distribution of the data, the ANOVA was performed within a nonparametric bootstrap procedure (Glick et al., 2014).

After the descriptive analysis was completed, the approach to translating outcomes into dollars was considered. The approach is fairly straightforward in that outcome variables are multiplied by a MCF, which represents the unit cost per outcome. Defining appropriate MCFs is where the challenge lies, although several sources of data can be useful for this purpose including published studies, government reports, and national data sets. For self-reported criminal activity, we use estimates of the cost per offense from McCollister et al. (2010), Blincoe et al. (2015), and Scott and Dedel (2006) (Blincoe et al. 2015; K. McCollister et al. 2010; Scott and Dedel, 2006). McCollister et al. (2010) used national data on criminal victimizations, arrests, and government crime prevention and prosecution expenditures to estimate the societal cost of 13 offenses, comprising victim costs, criminal justice system costs, and lost productivity among incarcerated offenders. Victim costs include medical and property costs, as well as the costs associated with pain and suffering and risk of homicide for a subset of more serious crimes. The cost per act of driving under the influence (not resulting in injury) comes from a study by Blincoe et al. (2015), which used data from the National Highway Traffic Safety Association (NHTSA) to estimate average unit costs of alcohol-involved automobile crashes for different levels of injury including fatalities. For prostitution, we apply the estimated transaction cost for the average prostitution act (~$50) based on a study of street prostitution by the U.S. Department of Justice (Scott and Dedel, 2006). We also used the distribution of offenses within four crime categories to generate a weighted average “cost per public order offense,” “cost per violent offense,” “cost per property offense,” and “cost per entrepreneurial offense.” A table of selected monetary conversion factors in 2016 dollars and data sources for criminal offenses, arrests, convictions, and incarceration is provided in the Appendix (Table 5).

To illustrate the process of translating outcomes into dollars, we applied MCFs to two of our measures: self-reported criminal activity (by crime category) and days of incarceration (see Table 3). Following per-offense cost calculations in McCollister et al. (2010), we opted to use the societal cost per offense to value self-reported criminal activity. For incarceration, we relied on a study by Henrichson and Delaney (2012), which estimated the cost per day of incarceration using national data (Henrichson and Delaney 2012). These MCFs were multiplied by number of offenses per 30 days (by crime category) and number of days incarcerated per 30 days to calculate baseline crime/incarceration costs.

**Table 3:** Average costs of baseline criminal activity and incarceration

	BRIGHT 1 (*n* = 2405)	BRIGHT 2 (*n* = 100)	STRIDE 1 (*n* = 50)	STRIDE 2 (*n* = 109)	RETAIN (*n* = 360)	PACTo (*n* = 409)
Crime Category						
Violent	$513 ($8687)	$210 ($1035)	/	/	$1686 ($31,158)	$16,260 ($199,528)
Property	$625 ($6493)	$1955 ($14,468)	/	/	$218 ($1570)	$2550 ($19,072)
Public	$498 ($6051)	$10 ($100)	/	/	$26 ($166)	$1390 ($9092)
Enterprise	$83 ($3123)	$0 ($0)	/	/	$12 ($152)	$0 ($0)
Incarceration	$436 ($853)	$574 ($973)	$19 ($134)	$47 ($308)	$49 ($247)	$28 ($192)

Notes: Standard deviations in parentheses. Crimes included in violent crime category for monetization: robbery, rape/sexual assault, assault/aggravated assault, murder; crimes included in property crime category for monetization: larceny, motor vehicle theft, household burglary, stolen property, arson, vandalism; crimes included in public crime category for monetization: prostitution, driving under influence; crimes included in enterprise crime category for monetization: fraud and forgery. Monetary conversion factors (MCFs) for criminal activity come from the societal cost per offense reported in McCollister et al. (2010). MCFs for incarceration come from Henrichson and Delaney (2012). See Table 5 in Appendix for MCFs

/ = not applicable for STRIDE 1 and 2

## Results

All studies asked about the number of times arrested and days incarcerated over different timeframes (lifetime, past 30 days, past 90 days, past 6 months). All but two studies asked about criminal activity and types of offenses, regardless of whether the crime resulted in an arrest. Studies had low rates of missing observations for most criminal activity and incarceration measures (0% - 8%). Given that these data were taken from baseline assessments, responsiveness and completeness of data were generally high and loss to follow-up was not yet an issue. Examination of logical skips (e.g., reporting no arrests in the past 6 months followed by appropriate code representing no days spent in jail) and valid responses (reporting a conviction followed by reporting type of offense convicted for) showed that quality and consistency of baseline data across studies was high. One exception was the PACTo study, which had up to 50% missing on self-reported arrest variables due to these questions being added to assessments after initiation of participant enrollment.

**Fig. 1:**
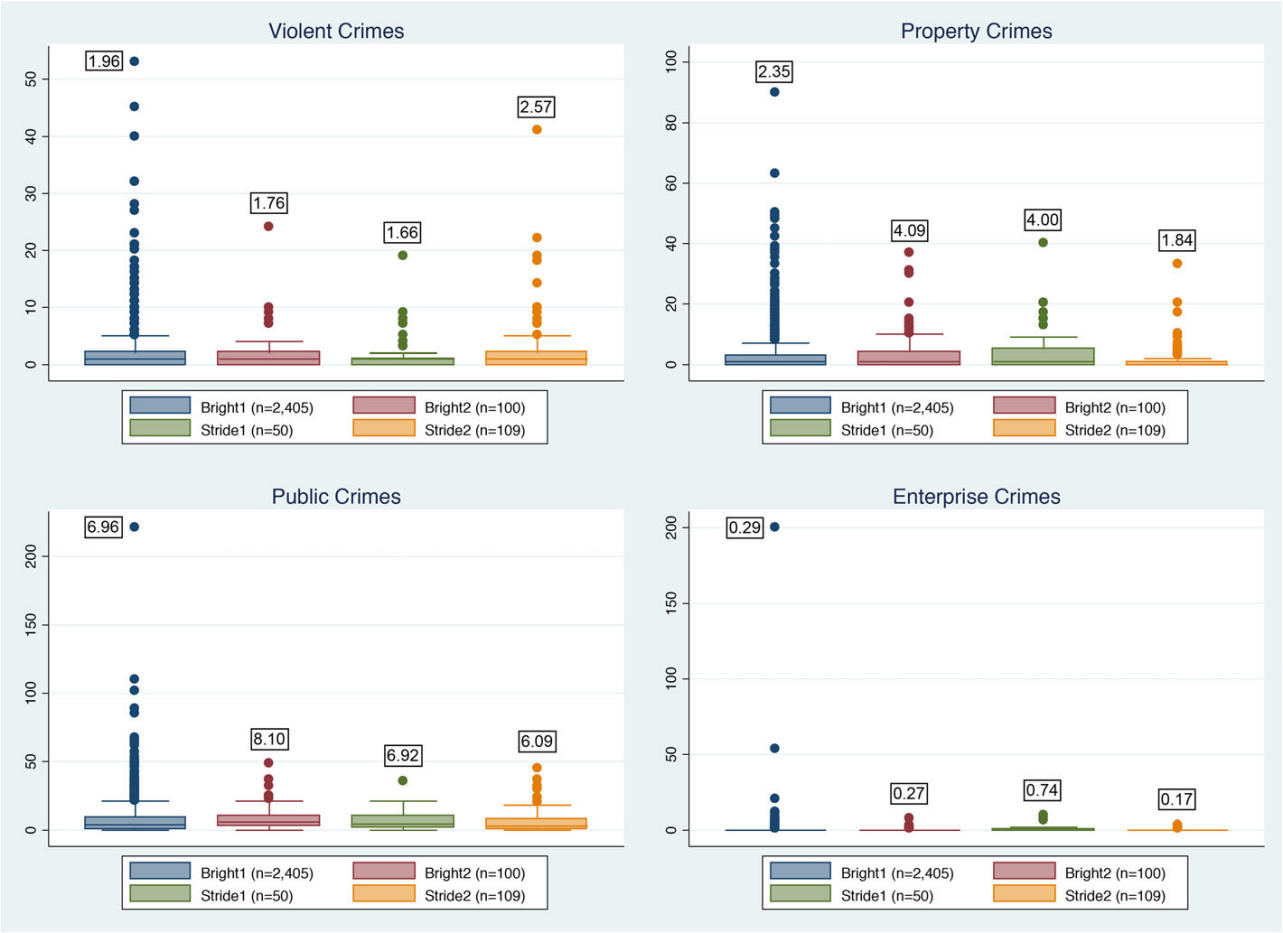
Lifetime arrests, by crime category. Means reported in boxes above the bar graphs. Means are tested using a bootstrap-based nonparametric one-way ANOVA. Means not statistically different (*p* < 0.5). BRIGHT 1 and 2 ask about arrests and charges separately. For this comparison, we assume a charge is equivalent to “arrested and charged.” The central box spans the first quartile to the third quartile; the whiskers above and below the box show 1.5 interquartile-range (IQR) from the corresponding quartile

Figure 1 illustrates the interquartile range of lifetime arrests and charges, by crime category. Four studies included this measure (BRIGHT 1 and 2, STRIDE 1 and 2). BRIGHT 1 and 2 asked about arrests and charges separately. For these comparisons, we assume a charge is equivalent to arrested and charged. Certain commonalities emerge from these groupings, namely that most respondents have been arrested for public order crimes (> 50%). Lifetime arrests for enterprise crimes and violent crimes are consistently low across all studies. The presence of large influential observations within violent crimes and property crimes is particularly notable for BRIGHT 1 and STRIDE 2. Means are statistically similar across studies based on a bootstrap-based nonparametric one-way ANOVA (*p* > 0.19).

**Fig. 2:**
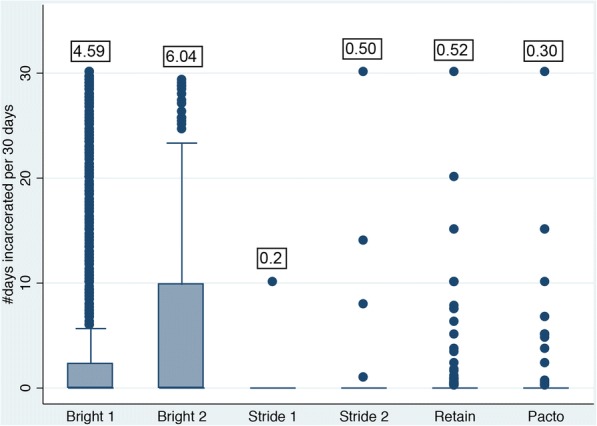
Average days incarcerated per 30 days. Means are displayed in boxes above bar graphs. Means tested using a bootstrap-based nonparametric one-way ANOVA; means statistically different (*p* < 0.001). STRIDE 1 and 2 report incarceration for past 30 days. The other studies report past 90 days or past 6 months, which were divided by 3 or 6 to represent an average 30 day estimate

Figure 2 shows the box plots and means of average days incarcerated per 30 days. BRIGHT 1 and 2, and RETAIN participants reported spending between 4 and 6 days per month incarcerated. The other studies had very low rates of incarceration (< 1 day per month). Means were statistically different across studies (*p* < 0.001) and all studies had influential observations (especially BRIGHT 1 and 2) falling well outside the interquartile range.

**Fig. 3:**
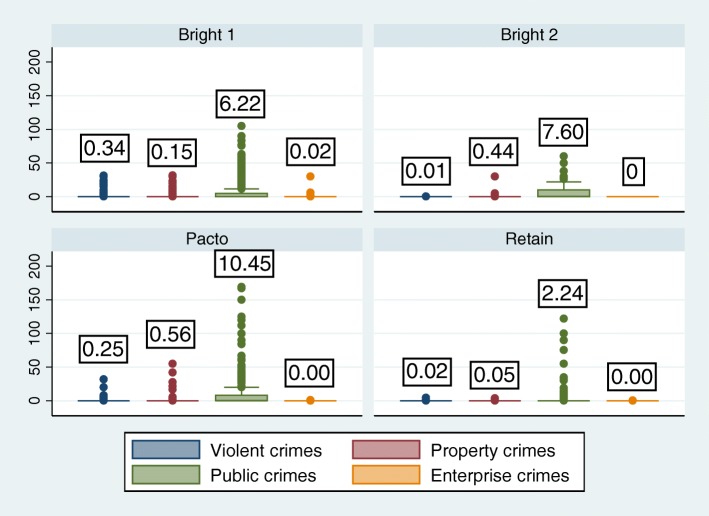
Average number of offenses committed per 30 days by crime category. Means are displayed in boxes above bar graphs. BRIGHT 1 and 2 ask about crimes committed in past 90 days, and RETAIN and PACTo ask about crimes committed in past 6 months. These values were divided to represent past 30 days for comparisons. Means tested using a bootstrap-based nonparametric one-way ANOVA; means not statistically different (*p* > 0.5). The central box spans the first quartile to the third quartile; the whiskers above and below the box show 1.5 interquartile-range (IQR) from the corresponding quartile

Self-reported criminal activity provides another perspective on criminal justice outcomes, given that not all offenses result in a criminal justice contact (i.e., an arrest, conviction, or incarceration). Four studies (BRIGHT 1 and 2; RETAIN, and PACTo) asked participants about the number of offenses committed for either the past 6 months or past 90 days, which we converted to average number of offenses committed per 30 days, contingent upon reporting any criminal activity at baseline. The data are presented in this manner because, on average, only 3% of respondents report committing any crimes. Figure 3 shows average offenses per 30 days, by crime category. Most individuals report committing public order crimes (> 50%). The most commonly reported offenses within this category were prostitution/pimping, larceny/shoplifting, and household burglary. Means for individual offenses were significantly different for violent offenses and public order offenses across studies (*p* < 0.001).

We also examined the mean number of criminal convictions by type of offense among those reporting any convictions for respondents in BRIGHT 1 and 2 (Table 4). Approximately 28% of BRIGHT 1 participants reported a conviction for larceny/shoplifting and, on average, they reported 3.4 lifetime convictions for this offense. Thirty percent of BRIGHT 2 participants reported being convicted for assault/aggravated assault/battery with a mean number of lifetime convictions of 1.0. In both studies, the highest percentage of lifetime convictions was for probation/parole violations (42%–51%) and drug charges (59%, both studies).

**Table 4:** Convictions at Baseline. Percent reporting any convictions in lifetime and mean number of times convicted by type of offense

Crime	BRIGHT 1 *(n = 2405)*		BRIGHT 2 *(n = 100)*	
	Percent reporting any conviction	Mean number of times convicted if reporting 1+ conviction	Percent reporting any conviction	Mean number of times convicted if reporting 1+ conviction
Property Offenses				
Fencing (buying or selling stolen property)	8.0%	1.9	1.0%	–
Burglary/attempted burglary/breaking and entering (home, auto, business)	19.0%	2.2	24.0%	1.0
Larceny and shoplifting	28.0%	3.4	38.0%	7.1
Vandalism/property damage/tagging	10.0%	1.5	11.0%	0.8
Arson (started a fire)	1.0%	1.0	3.0%	0.0
Auto theft	15.0%	2.0	17.0%	0.4
Car jacking	1.0%	1.3	0.0%	–
Public Order Offenses				
Prostitution or pimping	4.0%	4.1	9.0%	1.0
Probation/parole violations	42.0%	2.8	51.0%	3.4
Trespass of real property	10.0%	1.6	12.0%	1.4
Disorderly conduct	16.5%	2.5	7.0%	0.5
Drug charges (not drug dealing)	58.6%	3.0	59.0%	3.6
Drug dealing	40.2%	3.2	55.0%	2.6
Driving under the influence	9.6%	1.9	8.0%	1.1
Violent Offenses				
Robbery/attempted robbery/mugging	19.0%	1.6	18.0%	2.6
Assault/aggravated assault/battery	36.0%	2.4	30.0%	1.0
Homicide/manslaughter/attempted homicide	3.0%	1.0	2.0%	0.0
Weapons offenses	17.8%	1.4	17.0%	1.0
Sexual offenses (rape/aggravated assault/sex with a minor)	7.1%	1.0	3.0%	0.6
Enterprise Offenses				
Forgery	6.0%	1.8	8.0%	2.7
Fraud (bad checks, credit card fraud, etc.)	6.0%	1.4	5.0%	0.4

*Note*: sample size varies by offense (BRIGHT 2: N = 1–59), (BRIGHT 1: *N* = 26–1410)

Table 3 presents the average baseline costs of incarceration and criminal offending by crime category. As noted above, only the crimes that could be matched with a MCF were included in the calculation of crime costs. Public order crimes, representing the majority of reported offenses across all studies, had an average 30-day cost (per study participant reporting a crime) ranging from $10 (BRIGHT 2) to $1390 (PACTo). BRIGHT 2 also had the lowest average 30-day cost of violent crimes ($210); PACTo had the highest ($16,260). The average 30-day cost of property crime ranges from $220 (RETAIN) to $2560 (PACTo). Enterprise crime, which was rarely reported in these studies, had an average 30-day cost ranging from $0 (BRIGHT 2 and PACTo) to $80 (BRIGHT 1).

## Discussion

The objective of this study was to review criminal activity/criminal justice data available in six STTR studies and propose a set of standard measures that can be used for estimating the costs of criminal activity and criminal justice outcomes from different stakeholder perspectives. All six STTR studies had one measure in common, days incarcerated. At least four of these studies also collected data on arrests and criminal offenses. Based on our review of STTR studies, we recommend four crime/criminal justice measures for economic data harmonization projects: number of arrests, number of convictions, days of incarceration, and times committing criminal offenses, by type of offense. These measures are commonly assessed in other SUD research trials and observational studies and are therefore available in many existing datasets. In addition, criminal justice administrative databases typically track arrests and incarceration, and could serve as supplementary sources of economic data to support harmonization in SUD research.

Results summarized in the tables and figures illustrate the extreme variability in these crime/criminal justice measures. Criminal activity data are censored at zero and the distributions often have a long tail, where some observations lie well outside the interquartile range. It is important to note that the variation is partly due to systemic differences in police practices and supervision revocation across jurisdictions. These issues highlight the importance of statistical methods that account for the distribution of the observed data, and sampling uncertainty (Glick et al., 2014). If feasible, opportunities to pool individual data across studies and increase the sample size can moderate some of these concerns.

To account for extreme variability in most of these outcomes, grouping crimes by offense classification or calculating monthly averages per event facilitates more meaningful comparisons across studies. Adjusting for differences in assessment timeframe (recall timeframe) was relatively straightforward but raises a more general question about why assessment timeframes are so variable. This is perhaps most heavily influenced by funding limitations or other financial constraints. In other cases, interventions may be able to demonstrate clinical effectiveness over a shorter timeframe. From an economic perspective, having multiple follow-up assessments over a longer timeframe (e.g., data collected post-baseline at 6-, 12-, 18-, and 24-months) would be preferred for estimating changing costs over time. Future data harmonization initiatives should focus on timeframe harmonization as well.

These findings are meant to support the data harmonization efforts that are moving the SUD and HIV/HCV research fields forward (Brincks et al., 2017; Evans et al. 2010; Guerrero et al. 2015; Johnson et al. 2010; Niv et al., 2010). The methodological advantages of being able to synthesize and analyze data across multiple studies, such as improved statistical power and use of more sophisticated modeling approaches, is especially important for economic variables that tend to be skewed and often have higher rates of nonresponse, especially during follow-up assessments (Hussong, Curran, and Bauer, 2013; Brincks et al. 2017). A caveat is that pooling individual data from different studies is extremely complex and has been the subject of numerous methodological and applied studies (e.g., Friedenreich 1993; Curran et al., 2008; Brown et al., 2013; Bainter and Curran 2015). Identifying common measures across studies, as we have done here for estimating crime/criminal justice costs, informs data collection and harmonization efforts. It is just an initial step, however, in understanding the levels of variability within and across data, and evaluating threats to internal and external validity when formally merging data from multiple studies (Brincks et al. 2017).

For the criminal justice outcomes of arrests and convictions, and for certain offenses like probation/parole violations and drug law violations, monetary conversion factors are challenging to estimate and apply. We do not know, for instance, if an arrest results in an actual booking, time in jail, legal and adjudication processes, conviction, and incarceration. Reports have shown it is not possible to associate the number of recorded arrests with anticipated trajectories of criminal justice system costs using existing data. For example, a report by the New York State Attorney General on evaluating the *stop and frisk* law found that half of all arrests did not result in a conviction due to cases being dismissed or never prosecuted. Furthermore, less than 1% of arrests led to a jail or prison sentence and even fewer (0.1%) led to a conviction (New York Attorney General, 2013).

Evaluating the costs of probation or parole violations, drug offenses and other crimes present similar challenges, especially when relying solely on self-reported data. These are commonly reported crimes, but without knowing whether a violation resulted in a re-arrest or whether an act of prostitution or illicit drug sale resulted in an arrest, it is impossible to precisely calculate the criminal justice system costs attributable to these types of offenses. Additional studies are needed to improve the estimation of crime costs for a broader range of offenses and to identify administrative data sources that can be used to validate self-reported criminal activity and criminal justice contacts.

## Conclusions

The ability to estimate the value of reduced criminal activity provides a broader view on the potential economic impact of SUD and HIV/HCV interventions to inform resource allocation and public health policy. Recommendations on the conduct of cost-effectiveness and cost-benefit analysis highlight the relevance of different analytic perspectives for evaluating costs (Drummond et al., 2015; Neumann et al. 2016). For instance, from the public payer perspective, the tangible costs to the criminal justice system (e.g., policing, legal/adjudication, corrections) would be most relevant for an economic analysis; the societal perspective would look more broadly at victim costs as well. The opportunity to provide estimates of economic benefits or cost savings through reductions in crime/criminal justice contacts helps to provide a context for clinical findings of effectiveness for policy makers and other stakeholders who ultimately must choose what public health programs to support with limited taxpayer-funded budgets.

## Appendix

**Table 5:** Monetary conversion factors for criminal offenses, arrests, and incarceration (2016 dollars)

Criminal Offenses	Conviction Cost^a^	Societal Cost^a^
Murder	$447,281	$10,086,337
Rape/Sexual assault	$30,186	$270,352
Aggravated assault	$15,763	$120,166
Robbery	$9850	$47,507
Arson	$4705	$23,695
Motor vehicle theft	$4409	$12,095
Stolen property	$3283	$8953
Household burglary	$5007	$7256
Embezzlement	$5249	$6153
Forgery and counterfeiting	$4984	$5912
Fraud	$5495	$5650
Vandalism	$7800	$5457
Larceny/theft	$4742	$3966
Driving under the influence (no injury)^b^		$3130
Prostitution ^c^		$50
Incarceration and arrest		
Incarceration (day)^d^	$95	
Arrest (per event)^a^	$2407	

^a^McCollister et al. (2010)

^b^Blincoe, LJ., Miller, TR., Zaloshnja, E., Lawrence, BA. The Economic and Societal Impact Of Motor Vehicle Crashes, 2010 (Revised). 2015. Retrieved from https://crashstats.nhtsa.dot.gov/Api/Public/ViewPublication/812013

^c^Michael S. Scott and Kelly Dedel, “Street Prostitution,” Second Edition, United States Department of Justice, Office of Community Oriented Policing Services, November 28, 2006

^d^Henrichson and Delaney (2012)

Notes: Conviction costs represent the total cost to the criminal justice system (policing, legal and adjudication, corrections) based on McCollister et al. (2010). Societal costs include direct victim costs, victim pain and suffering, risk of homicide costs, and criminal justice system costs. Based on McCollister et al. (2010). Cost per arrest is based on national policing expenditures

## Abbreviations

ANOVA: Analysis of variance
HCV: Hepatitis C virus
HIV: Human immunodeficiency virus
NIDA: National Institute on Drug Abuse
STTR: Seek, Test, Treat and Retain
SUDs: Substance use disorders
